# An untargeted metabolomic approach to investigate antiviral defence mechanisms in memory leukocytes secreting anti-SARS-CoV-2 IgG in vitro

**DOI:** 10.1038/s41598-022-26156-4

**Published:** 2023-01-12

**Authors:** Gevi Federica, Fanelli Giuseppina, Lelli Veronica, Zarletti Gianpaolo, Tiberi Massimo, De Molfetta Veronica, Scapigliati Giuseppe, Timperio Anna Maria

**Affiliations:** 1 Department of Ecological and Biological Sciences, University of Tuscia, 01100 Viterbo, Italy; 2 Department of Innovative Biology, Agro-Food and Forestry, University of Tuscia, 01100 Viterbo, Italy

**Keywords:** Biochemistry, Diseases

## Abstract

Evidence shows that individuals infected by SARS-CoV-2 experience an altered metabolic state in multiple organs. Metabolic activities are directly involved in modulating immune responses against infectious diseases, yet our understanding of how host metabolism relates to inflammatory responses remains limited. To better elucidate the underlying biochemistry of the leukocyte response, we focused our analysis on possible relationships between SARS-CoV-2 post-infection stages and distinct metabolic pathways. Indeed, we observed a significant altered metabolism of tryptophan and urea cycle pathways in cultures of peripheral blood mononuclear cells obtained 60–90 days after infection and showing in vitro IgG antibody memory for spike-S1 antigen (n = 17). This work, for the first time, identifies metabolic routes in cell metabolism possibly related to later stages of immune defence against SARS-CoV-2 infection, namely, when circulating antibodies may be absent but an antibody memory is present. The results suggest reprogramming of leukocyte metabolism after viral pathogenesis through activation of specific amino acid pathways possibly related to protective immunity against SARS-CoV-2.

## Introduction

Coronavirus disease 19 (COVID-19) is an acute illness caused by SARS-CoV-2 (severe acute respiratory syndrome coronavirus 2) with initial clinical symptoms such as cough, fever, malaise, headache, and anosmia^1^. This viral infection stimulates immune responses directed against the spike protein (S1 protein) present on the surface of SARS-CoV-2, which is a ligand that binds to angiotensin-converting enzyme 2 (ACE2) receptors on host cells. Attachment of the SARS-CoV-2 spike glycoprotein with angiotensin-converting enzyme 2 (ACE2), as its cellular receptor, triggers complex molecular events that lead to hyperinflammation^2^.

Under severe conditions, excessive and uncontrolled production of pro-inflammatory cytokines, including IL-6, IL-1, and TNF-α, results in convergence of immune cells and leads to a systemic inflammatory response known as a cytokine storm^3^.

Of note, among elevated inflammatory mediators, blood IL-6 levels, known as the IL-6 amplifier, correlate highly with the lethal complications of COVID-19, suggesting that fatal COVID-19 can be characterized as a cytokine release syndrome (CRS) induced by a cytokine storm, with high mortality^4^. IL-6 binds the IL-6 receptor, and the latter recruits JAK, which transduces a cascade signal to activate signal transducer and activator of transcription 3 (STAT3), which participates in the induction of inflammatory responses during coronavirus infection. The IL-6/JAK/STAT-3 axis potently contributes to the pathogenesis of COVID-19^5^. Indeed, this signalling pathway exerts pleiotropic impacts of IL-6 on innate (macrophages, neutrophils and natural killer cells) as well as specific (T and B cells) immune cells, supporting the cytokine storm^6^. Secretion of such cytokines and chemokines attracts immune cells, notably monocytes and T lymphocytes, from the blood into the site of infection^7^. In the respiratory tract, recruitment of immune cells and lymphocytes from the blood might explain the lymphopenia and increased neutrophil–lymphocyte ratio seen in approximately 80% of patients with SARS-CoV-2 infection^8^, and after contact with viral antigens, most effector T cells undergo apoptosis. Next, a pool of memory T cells is formed to fight reinfection. In the case of a subsequent infection, CD4^+^ memory T cells become restimulated and activate B cells and other immune cells by producing cytokines with the help of the CD8^+^ memory T cells that kill virus-infected cells^9,10^.

Among host responses, metabolic changes and differences in leukocyte composition can be found during various stages of SARS-CoV-2 infection, suggesting that monocytes, neutrophils and T-lymphocytes are associated with the onset and progression of COVID-19 infection^11^. Increased signals of monocytes, dendritic cells and the mitochondrial respiratory electron transport chain in SARS-CoV-2 infection suggest a critical role for metabolic pathways in the immune response of COVID-19 patients^12^.

Because immune responses are tightly connected to metabolic programmes^13,14^, multiple approaches are currently being used to identify key pathways involved in SARS-CoV-2. In particular, altered tryptophan metabolism involved in kynurenine regulating inflammatory and immunity pathways has been identified through targeted and untargeted metabolomic analyses^14^. Moreover, cytosine (reflecting viral load), kynurenine (reflecting host inflammatory response), nicotinuric acid, and multiple short chain acylcarnitines (energy metabolism) are altered, in agreement with a severe outcome of the pathology, as already reported^15^.

A recent study also revealed that metabolites involved in arginine metabolism, including glutamate, arginine, N-(l-arginino)-succinate, citrulline, ornithine, glutamine, 2-oxo-glutarate, N-acetyl-L-glutamate, and urea, in the sera of COVID-19 patients are significantly decreased compared with in healthy controls but that their levels were either unchanged or even increased in the sera of non-COVID-19 patients, suggesting massive metabolic suppression in severe COVID-19 patient sera^16^. Therefore, serum metabolism of patients with COVID-19 is specifically altered, and understanding the associations of these alterations with COVID-19 development is necessary to identify potential biomarkers and models for distinguishing COVID-19 patients from healthy controls (HCs) and suspected patients^17^.

**Figure 1:**
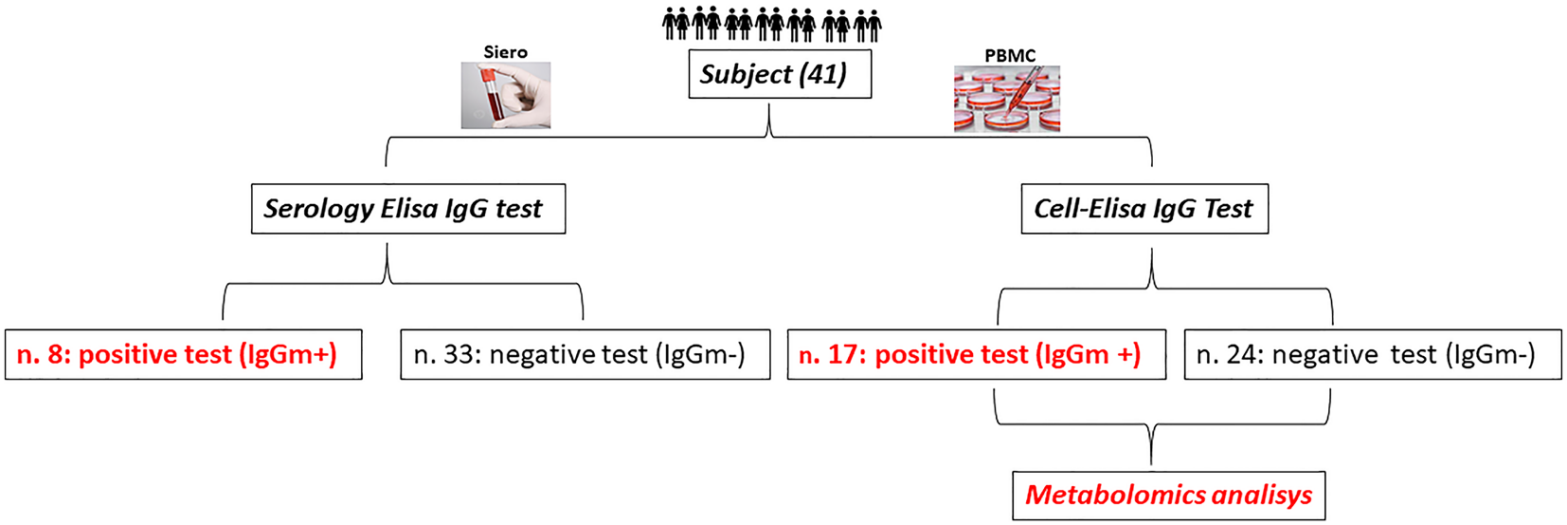
Clinical characteristics of the enrolled subjects. Samples were tested using both the ELISA serological test and In-Cell ELISA. In the first case, 8 subjects showed serological positivity; through In-Cell ELISA, we identified 17 positive samples for in vitro specific IgG secretion (IgGm^+^). Therefore, 9 individuals who had negative ELISA serological test results were positive by In-Cell ELISA.

Despite increasing knowledge of metabolic changes in COVID-19 patients, little attention has been given to post-infection stages (> 60 days) in which immune memory becomes responsible for protecting against SARS-CoV-2 reinfection^18^. Indeed, as with other viral pathologies, such as severe acute respiratory syndrome (SARS) and Middle East respiratory syndrome (MERS), there is concern about COVID-19 effects, which may have long-term consequences, and the criteria for “recovery” are mainly focused on the reduction of respiratory symptoms^19^. In this context, knowledge of possible metabolic pathways linked to leukocytes and related to later stages of antiviral defences may be of considerable importance. In this study, we performed metabolomic profiling coupled with multivariate statistical analysis obtained from 41 cell cultures of peripheral blood mononuclear cells (PBMCs), 17 of which displayed an in vitro IgG memory for spike-S1 antigen 60–90 days after infection, as determined by In-Cell ELISA^20^. We focused our attention on in vitro antibody memory cells, and we noted that leukocytes from donors responded efficiently in In-Cell ELISA (see Fig. 1). By using our method, which is reliable, unambiguous and accurate, we found an alteration and involvement of amino acid pathways, which we presume to constitute a protective mechanism over time against SARS-CoV-2.

## Materials and methods

### Subject recruitment

Forty-one subjects (23 males and 18 females, Table 1) undergoing COVID-19 serological analysis (Centro Polispecialistico Giovanni Paolo I, Viterbo, I) were enrolled in this study from October 2020 to March 2021. All participants provided informed written consent to participate in the research project, and the study was approved by the Regional Ethical Board of IRCCS Lazio in Ospedale L. Spallanzani, Roma (number 169, approval 22/07/2020). In accordance with the Helsinki Declaration, written informed consent was obtained from all subjects. Subjects with comorbidities were excluded on the basis of the declaration released by donors when enrolled in the protocol. No specific medical analyses were performed, with the exclusion of COVID-19 screening for declared asymptomatic donors 3 days before blood draw.

**Table 1:** *In-Cell ELISA absorbance (A450 nm) net values; positive values are in bold, with a cut-off of 0.07.

Age/Gender	In vitro IgG memory	Days after first screening
	(Cell-ELISA)*	
33 F	0.039	78
33 F	**0.239**	92
33 M	0.023	35
35 M	0.02	35
36 M	**0.07**	64
36 F	**0.292**	92
39 F	**0.223**	88
40 F	**0.291**	93
40 M	0.019	35
40 M	0.026	35
42 F	**0.258**	92
42 F	**0.087**	92
42 M	0.024	35
43 M	0.024	35
44 M	0.023	35
44 M	0.02	35
45 M	0.023	35
45 M	0.017	35
46 M	0.058	35
47 F	**0.293**	92
47 F	**0.085**	93
49 M	0.021	35
49 M	0.022	35
49 M	0.025	35
50 M	0.019	35
50 F	**0.141**	93
53 M	0.067	35
54 M	0.014	35
57 F	**0.177**	94
58 F	**0.245**	88
63 M	0.038	35
67 F	**0.285**	95
68 F	0.05	35
70 F	**0.118**	93
71 M	**0.082**	80
72 M	0.023	35
72 M	0.033	35
72 F	0.027	35
72 F	**0.116**	93
73 M	0.037	35
74 F	**0.217**	93

The In-Cell ELISA protocol and cut-off value were as previously described^20^. Materials were obtained from a commercial kit (Dia. Pro Diagnostics Bioprobes srl, Milano, Italy. Lot 0420/5AA, Ref: COV19G.CE.192). The negative controls were asymptomatic donors screened for COVID-19 at 35 and 3 days before In-Cell ELISA.

The demographic and clinical characteristics of the enrolled subjects are shown in Fig. 1.

### Cell-ELISA and PBMC cultures

Determination of in vitro antibody memory was performed by measuring specific IgG produced by PBMCs incubated in wells coated with the spike-S1 protein, employing an In-Cell ELISA previously described^20^. S1 protein was used as the antigen, as supplied by a commercial ELISA kit (Dia. Pro Diagnostics Bioprobes srl, Milano, Italy. Lot 0420/5AA, ref: COV19G.CE.192). The individuals were enrolled on the basis of a serology assays performed 60–90 days before to match the In-Cell ELISA timing, a timing selected to be beyond a natural serum IgG decay^20^. Spike-S1 is widely employed either as an antigen in vaccines or in immunoenzymatic/in vitro assays. None of the enrolled subjects had been vaccinated when the assays were performed.

Parallel cultures of untreated PBMCs employed in In-Cell-ELISA were incubated in microplates (Costar, USA) and then centrifuged at 500xg. The pelleted cells were immediately employed for metabolomic analysis.

### Metabolite extraction and UHPLC‒MS analysis

Metabolites in the PBMC samples were extracted in technical triplicate, Each sample was added to 1000 μl of a chloroform/methanol/water (1:3:1 ratio) solvent mixture stored at − 20 °C. The tubes were mixed for 30 min and subsequently centrifuged at 1000 × g for 1 min at 4 °C before being transferred to − 20 °C for 2–8 h. The solutions were then centrifuged for 15 min at 15,000 × g and dried to obtain visible pellets. The dried samples were resuspended in 0.1 mL of water and 5% formic acid and transferred to glass autosampler vials for LC/MS analysis. Twenty microlitres of extracted supernatant sample was injected into an ultrahigh-performance liquid chromatography (UHPLC) system (Ultimate 3000, Thermo) run in positive mode; a Reprosil C18 column (2.0 mm × 150 mm, 2.5 μm-DrMaisch, Germany) was used for metabolite separation. For positive ion mode (+) MS analyses, a 0–100% linear gradient of solvent A (ddH2O, 0.1% formic acid) to B (acetonitrile, 0.1% formic acid) was employed over 20 min, returning to 100% A in 2 min and holding solvent A for a 1-min post time hold. Acetonitrile, formic acid, and HPLC-grade water and standards (≥ 98% chemical purity) were purchased from Sigma Aldrich. Chromatographic separations were performed at a column temperature of 30 °C and a flow rate of 0.2 ml/min. The UHPLC system was coupled online with a Q-Exactive mass spectrometer (Thermo) scanning in full MS mode (2 μ scans) at a resolution of 70,000 in the 67 to 1000 m/z range, a target of 1106 ions and a maximum ion injection time (IT) of 35 ms with 3.8 kV spray voltage, 40 sheath gas and 25 auxiliary gas. Calibration was performed before each analysis against positive or negative ion mode calibration mixtures (Pierce, Thermo Fisher, Rockford, IL) to ensure error of the intact mass within the sub-ppm range.

### Metabolomic data processing and statistical analysis

Raw files of replicates were exported as .mzXML files and processed through MAVEN.8.1. Mass spectrometry chromatograms were created for peak alignment, matching and comparison of parent and fragment ions with tentative metabolite identification (within a 2-ppm mass-deviation range between the observed and expected results against an imported KEGG database). Fold change analysis was performed on the entire metabolomics dataset using MetaboAnalyst 5.0 software. Before the analysis, raw data were normalized by median and autoscaling to increase the importance of low-abundance ions without significant amplification of noise, and the fold change threshold was set to > 1.5. The purpose of fold change (FC) analysis was to compare absolute value change between two group averages and find some features that changed consistently (i.e. up-regulated or down-regulated) between the two groups. To analyse a correlation network of the compounds in shared pathways, MetScape, an app implemented in Java and integrated with Cytoscape (version 3.8.2), was used. To further explore the metabolic differences between the two groups of subjects, detailed analysis of altered metabolic pathways and networks was carried out using MetaboAnalyst 5.0 software. A web-based tool to identify biologically meaningful patterns in quantitative metabolomic data Metabolite Set Enrichment Analysis (MESA) included in the MetaboAnalyst platform was used to perform pathway analyses. The Kyoto Encyclopedia of Genes and Genomes (KEGG) human metabolic pathways library^21^ was employed for pathway analysis.

### Ethics approval and consent to participate

All participants provided informed written consent to participate in the research project, and the study was approved by the Regional Ethical Board in Ospedale L. Spallanzani, Roma (number 169, approval 22/07/2020). In accordance with the Helsinki Declaration, written informed consent was obtained from all subjects.

## Results

Forty-one subjects were enrolled in this observational study. Both ELISA serological tests and In-Cell ELISA tests were performed on the samples. In the first case, 8 subjects were positive based on serological analysis of anti-SARS-CoV-2 IgG; through the In-Cell ELISA assay, we identified 17 positive samples for in vitro specific IgG secretion (IgGm^+^). Comparison of these two tests showed that the 9 individuals with a negative result for the ELISA serological test were positive for the In-Cell ELISA and therefore appeared to have circulating B cells producing antibodies against SARS-CoV-2, which were probably undetectable in serum (Fig. 1). The remaining 24 samples were used as control samples, as they were untreated. PBMCs that had never encountered the antigen before. Hence, metabolomics analysis was performed on peripheral blood mononuclear cell (PBMc) cultures displaying an anti-spike-S1 IgG memory (IgGm^+^; N = 17) and untreated PBMCs (IgGm^−^; N = 24) by In-Cell ELISA at 60–90 days after SARS-CoV-2 screening.

### Pathway analysis

**Figure 2:**
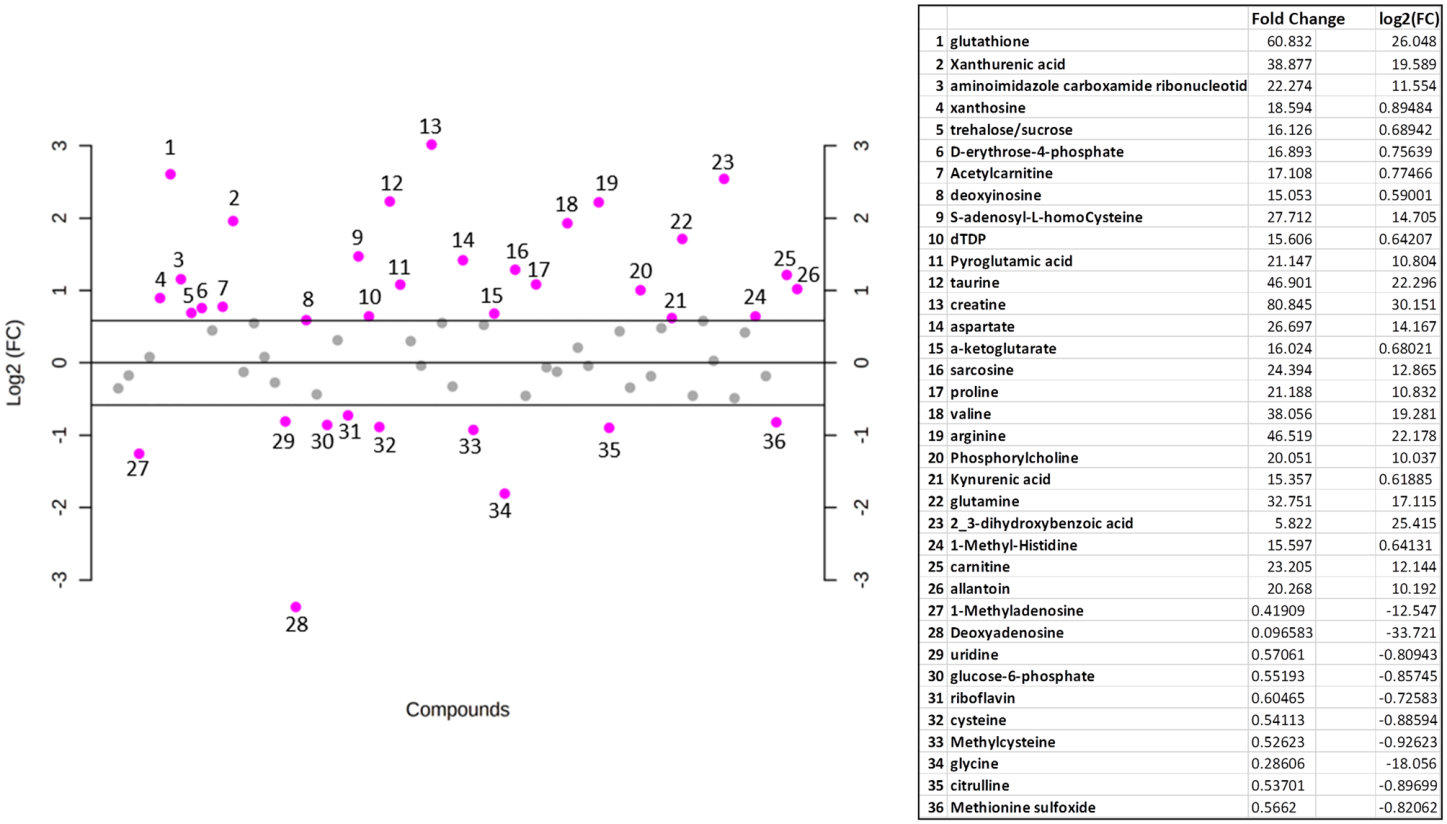
Fold change representation of all analysed metabolites. Data are log2-fold-change in relative abundance of untargeted metabolites in biological replicates of IgGm^+^ relative to IgGm^−^. Dashed lines indicate a log2-fold-change of |1|. Purple dots indicate metabolites with >|1| log2-fold-change, and grey dots represent metabolites with <|1| log2-fold-change. The table represents 26 metabolites with the most significant changes in IgGm^+^; 10 metabolites resulted in an increase in IgGm^−^.

We used MetaboAnalyst 5.0 platforms to perform untargeted metabolomics to detect possible alterations in relevant metabolites in IgGm^+^ on the basis that the two groups were well clustered by PLS-DA, as already reported^22^. Fold change analysis was used to identify metabolites altered in IgGm^+^ and involved in defence mechanisms against COVID-19, with an FC value greater than 1.5 considered a significant threshold. The fold change value for each metabolite was log2 transformed, and the corresponding p value was − log10 transformed. Based on these IgGm^+^ results, 26 metabolites showed the most significant changes in IgGm^+^, whereas 10 metabolites resulted in an increase in IgGm^−^ (Table 1 and Fig. 2).

**Figure 3:**
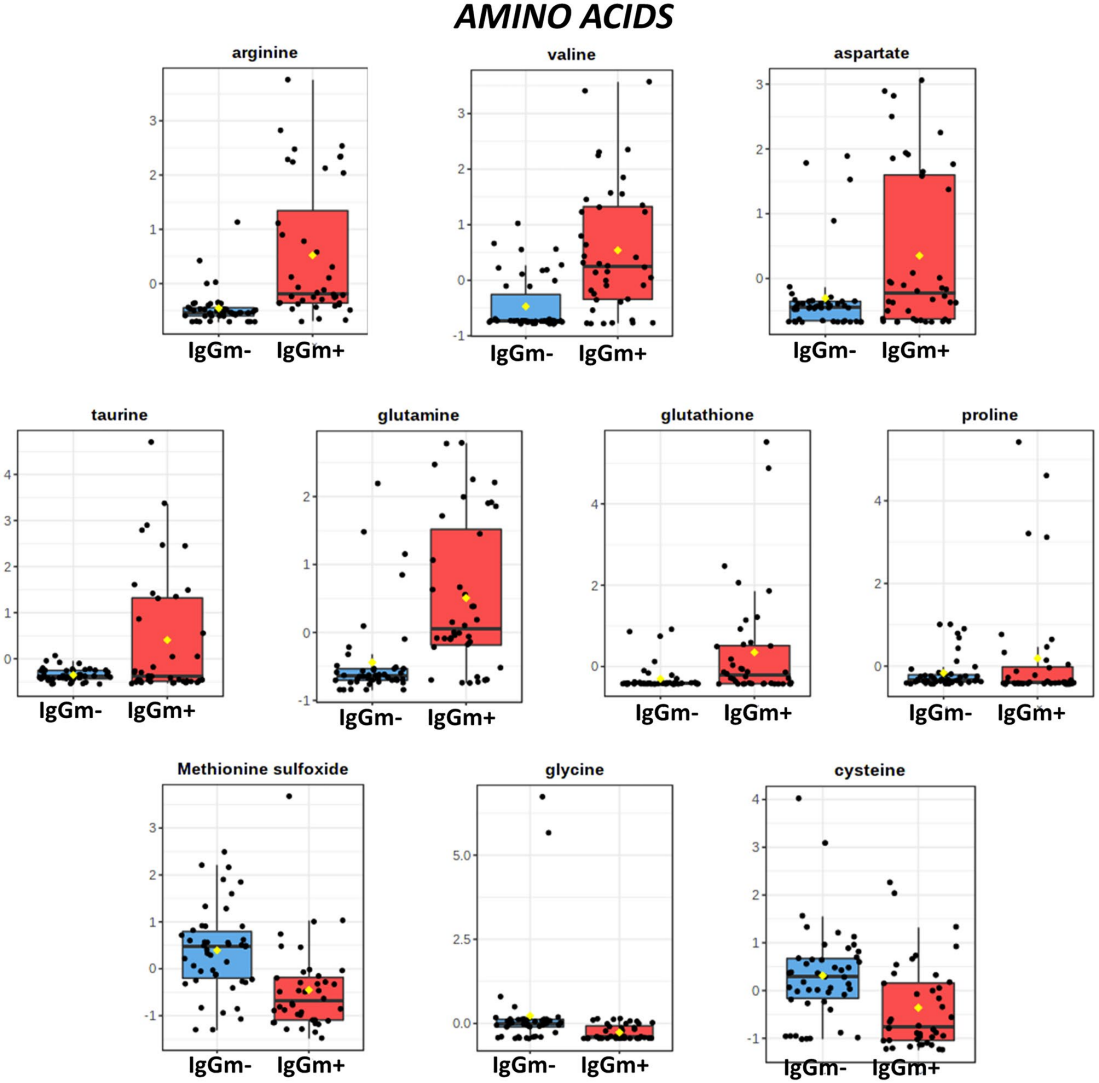
Amino acid levels in PBMC samples. Bar plots show the mean and standard deviation (SD) of the normalized peak area.

This analysis revealed that of the 36 significantly altered metabolites, COVID-19 had the greatest effect on amino acid metabolism. Namely, gluconeogenic amino acids (e.g., arginine, valine, glutathione, aspartate, proline and glutamine) and taurine (sulfur-containing amino acid) were up-regulated in IgGm^+^ cells. In contrast, cysteine, glycine and methionine sulfoxide (oxidized forms of sulfur-containing amino acids) showed a decrease (Fig. 3).

Based on the online database of metabolic pathways (KEGG, http://www.genome.jp/kegg/), MetScape software running on Cytoscape was used to visualize and interpret metabolites in the context of a global metabolic network (Supplementary Fig. 1). Network visualizations may be helpful to show connections between metabolites and to understand relationships between compounds and pathways to obtain a high-level overview of changes in metabolic activities caused by COVID-19 IgGm^+^ . The network reflects the complexity of the effects of the pathology and provides further evidence for the involvement of tryptophan metabolism, the urea cycle and metabolism of arginine, proline, glutamate, aspartate and asparagine.

**Figure 4:**
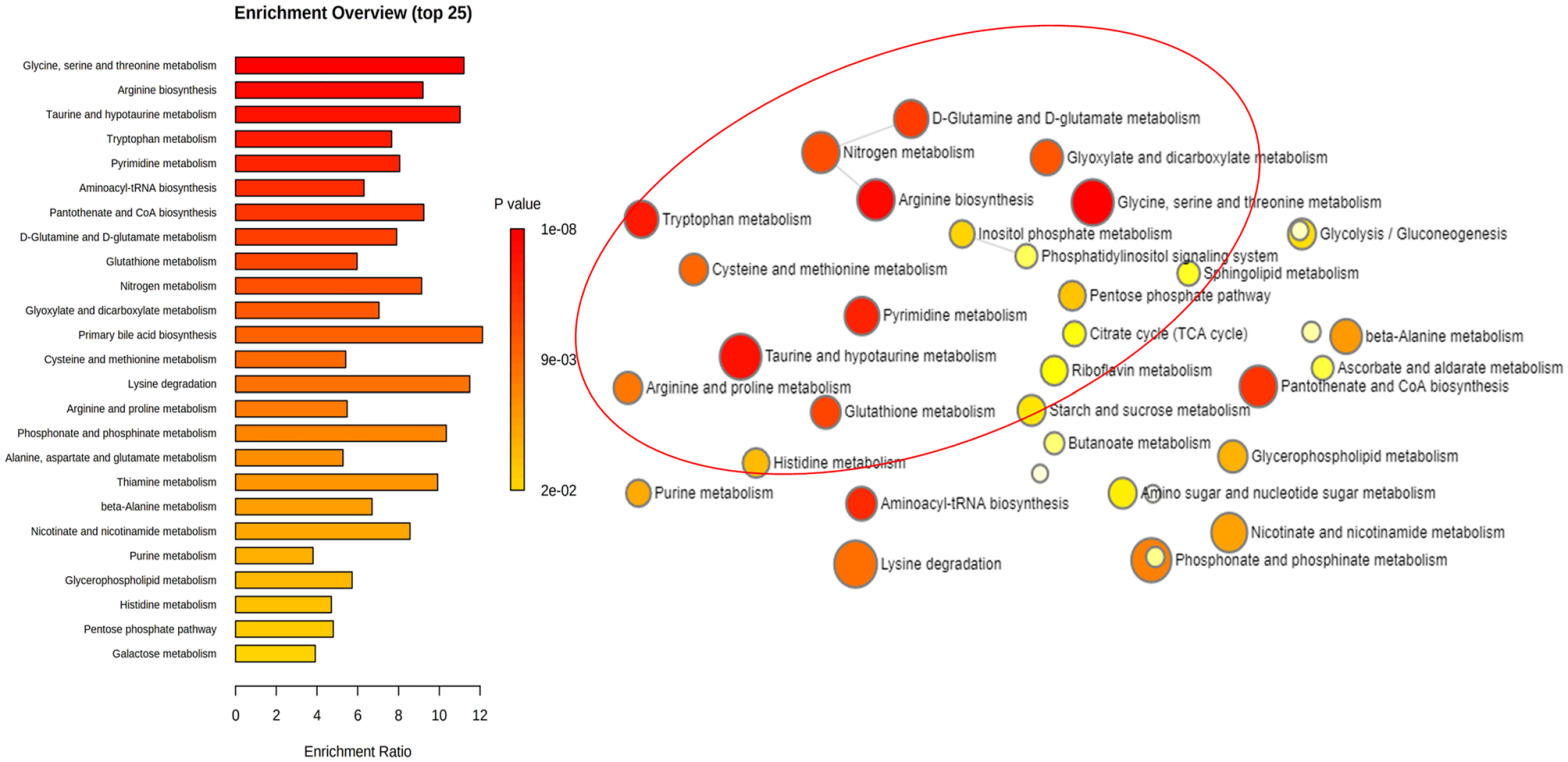
Pathway enrichment analysis of untargeted metabolomics. Significant alteration in leukocyte IgGm^+^ on amino acid metabolism, especially pathways involved in glycine serine threonine metabolism, arginine biosynthesis, and tryptophan metabolism.

**Figure 5:**
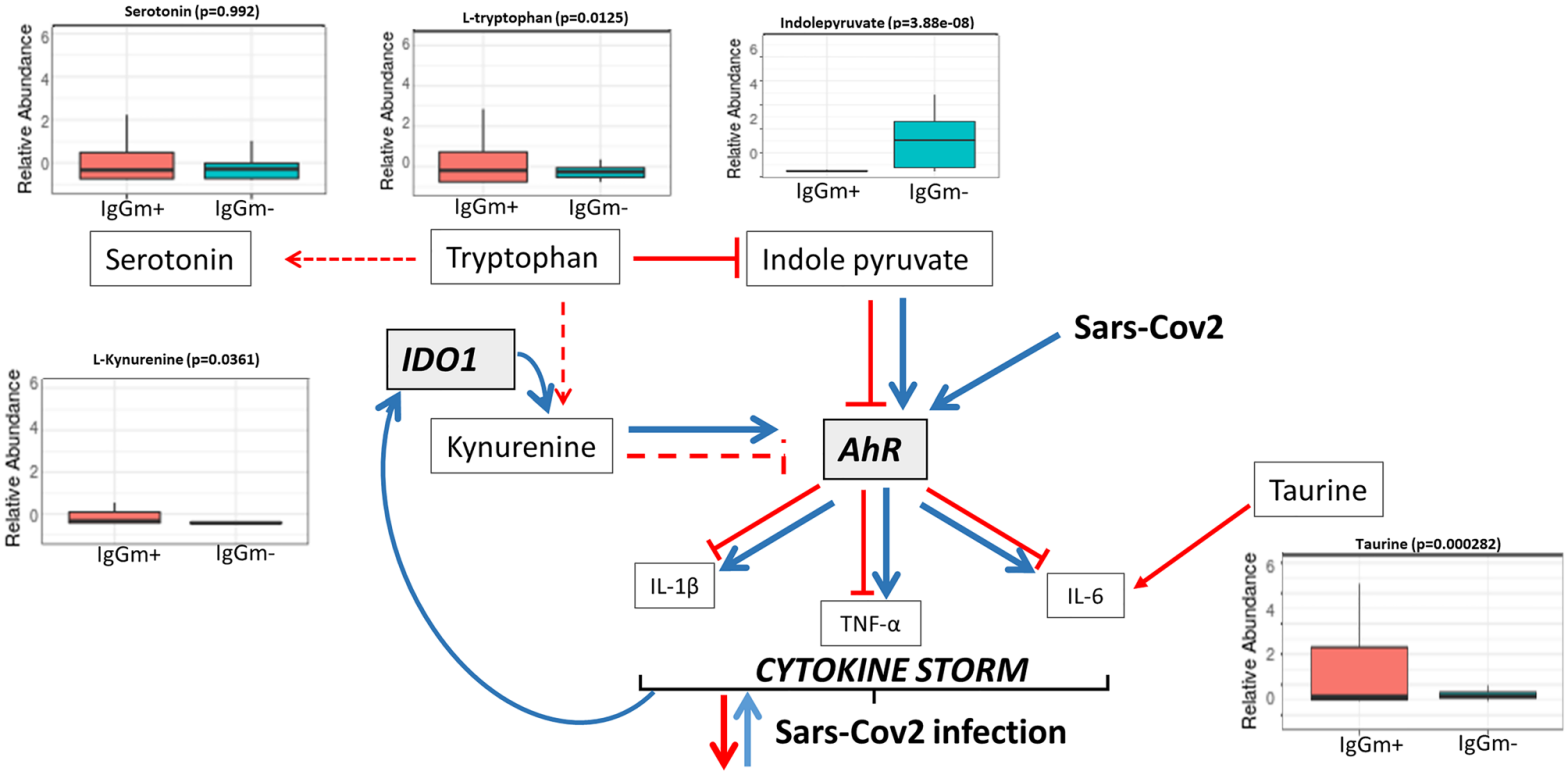
A schematic diagram of tryptophan metabolism, including box plots of quantified metabolites. Data are expressed as the mean and standard deviation (SD) of the normalized peak area. Reduction in the level of indolepyruvate and increase in the level of kynurenine act on AhR signalling pathways contribute to defence against SARS-CoV-2 infection.

**Figure 6:**
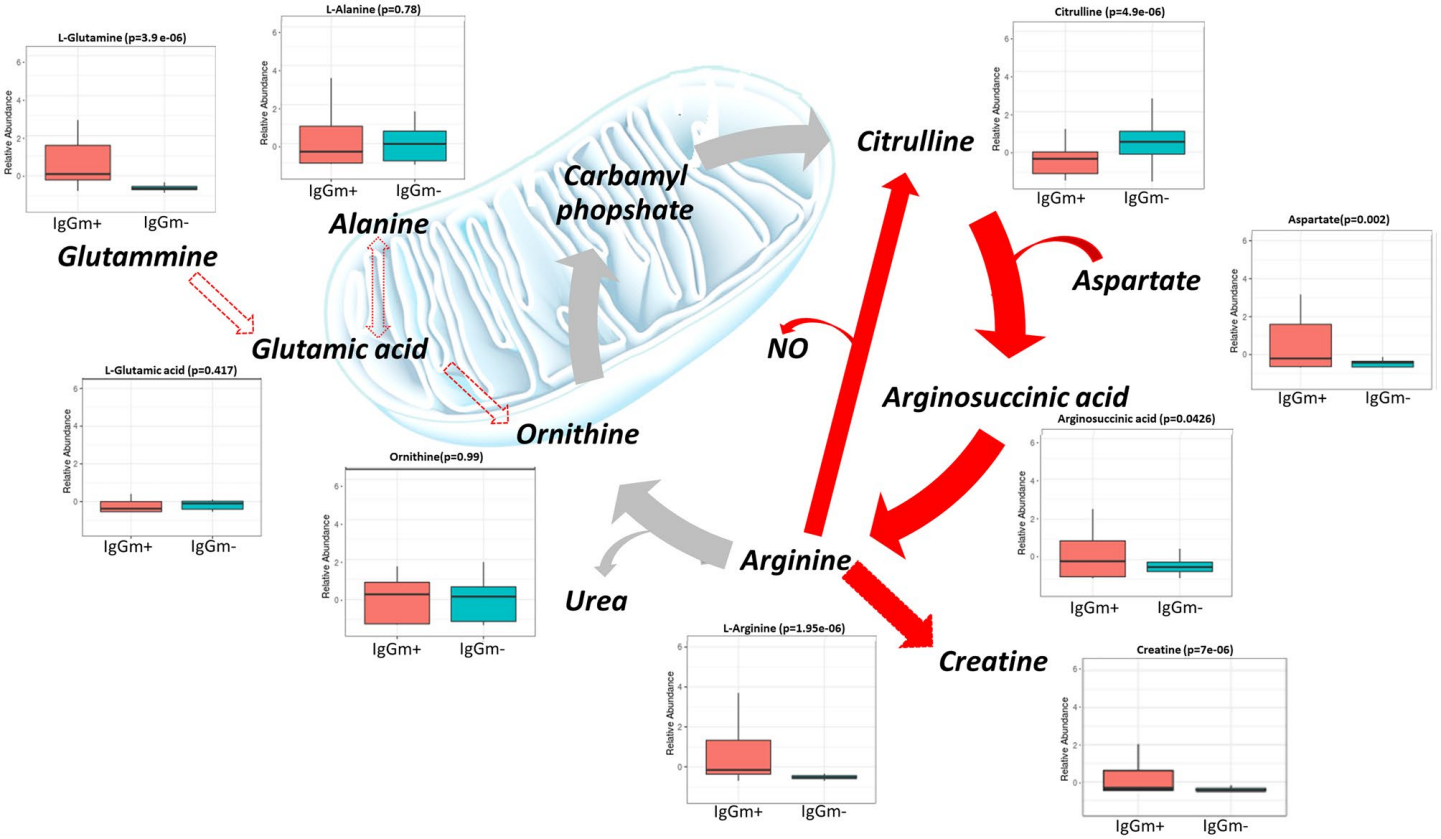
Schematic representation of the urea cycle and its connected metabolism, including box plots of quantified metabolites. Data are expressed as the mean and standard deviation (SD) of the normalized peak area. The urea cycle is associated with the citrulline NO pathway (in red lines); ornithine, glutamic acid and alanine, but not glutamine, did not change in abundance, suggesting altered homeostasis of the urea cycle.

Complementary to the network analysis, pathway enrichment analysis revealed a significant alteration in leukocyte IgGm^+^ on amino acid metabolism, especially pathways involved in glycine serine/threonine metabolism, arginine biosynthesis, and tryptophan metabolism (Fig. 4). Top hits from these pathways were mapped against KEGG pathways, and tryptophan metabolism and arginine biosynthesis (urea cycle) are highlighted in Figs. 5, 6.

### Tryptophan metabolism and arginine biosynthesis

Tryptophan metabolism was one of the top pathways altered in leukocytes displaying IgG antibody memory to SARS-CoV-2. Specifically, tryptophan increased in IgGm^+^ and serotonin did not undergo significant change compared with controls (Fig. 5). At the same time, the reduction in the level of indolepyruvate and the increase in the level of kynurenine, which are known to be rich sources of aryl hydrocarbon receptor (AhR) ligands, suggested restoration of the kynurenine pathway to decrease the cytokine storm, which was confirmed by the increased level of taurine in IgGm^+^ cells. In addition to tryptophan metabolism, arginine biosynthesis has a significant impact on leukocytes showing IgGm^+^ . Some metabolites of the urea cycle (e.g., arginine, aspartate and arginosuccinic acid) were increased in IgGm^+^ samples, leading to up-regulation of creatine. In contrast, ornithine, glutamic acid and alanine, but not glutamine, did not change in abundance, suggesting altered homeostasis of the urea cycle (Fig. 6). Consequently, citrulline decreased in IgGm^+^ samples because it is not produced by ornithine and carbamoyl phosphate but is converted to arginosuccinic acid.

## Discussion

Although host defence mechanisms against SARS-CoV-2 infection are still poorly described, they are critically important in shaping the disease course and possible outcome. An untargeted metabolomics profile can integrate the poor knowledge of the molecular mechanisms underlying the infection and the reprogramming of leukocyte metabolism after viral pathogenesis. The present study provides the first metabolic characterization of PBMC IgG memory after SARS-CoV-2 infection. The results show marked alterations in metabolism of amino acids, particularly tryptophan and arginine.

Tryptophan (Trp) metabolism is one of the major pathways affected in IgGm^+^ leukocytes, as shown in our non-targeted metabolomic data results (Fig. 5). Trp fuels the synthesis of kynurenine (Kyn), serotonin (5-HT) and indoles. A recent study by Gardinassi et al. revealed high involvement of inflammatory networks and increased expression of genes involved in tryptophan metabolism in COVID-19 patients^23^. Interestingly, a decrease in tryptophan and a contemporary increase in the kynurenine pathway are the major effects of COVID-19, regulating inflammation and immunity^14^. The same result was obtained by Danlos et al.^23^ and these findings highlight that tryptophan tends to diminish but that its immunosuppressive metabolite kynurenine increases in critical care patients compared to mild cases. This metabolic pathway involves conversion of tryptophan to L-kynurenine by the enzymes indoleamine 2,3-dioxygenase 1 and 2 (IDO1 and IDO2). IDO enzymes are activated by inflammatory cytokines (IFN-α,-β,-γ, TNF-α), particularly interleukin-6 (IL-6)^24^. These changes in tryptophan metabolism due to regulation of IDO enzymes correlate with a greater increase in interleuknin-6 (IL-6) level^14^. Inducing a cytokine storm through aryl hydrocarbon receptor (AhR)^25^. At the same time, release of IFN-γ by monocytes, macrophages, and T cells following viral infection leads to up-regulation of IDO^26,27^, IFN-γ and kynurenine levels^28^, confirming an intertwined relationship in the inflammatory host response to SARS-CoV-2 infection^28^. The resulting ‘cytokine storm syndrome’ is perhaps one of the critical hallmarks of COVID-19 disease severity^29^.

According to our results, the increased level of tryptophan, an almost unchanged level of serotonin, and the strongly decreased level of indole pyruvate support the hypothesis of restoration of the kynurenine pathway through attenuation of IDO activity. This result is in agreement with the findings of Xiao et al. who showed that manipulation of tryptophan metabolism by an IDO1 inhibitor leads to a marked decline in proinflammatory cytokines, indicating its therapeutic potential in controlling CRS in SARS-CoV-2 infection^30^.

In addition to kynurenine, indole pyruvate is an endogenous ligand that activates AhR^31,32^. Therefore, the level of indole pyruvate reduction negatively modulates AhR activity in leukocytes that display IgGm^+^ for spike-S1 antigen at 60–90 days after infection. Moreover, the increased level of taurine in IgGm^+^ attenuates IL-6 levels^33^, cooperating to reduce inflammation. Increasing evidence from several studies shows that tryptophan can reduce inflammatory reactions and enhance the immune system^34^. Furthermore, recent studies have suggested a link between tocilizumab immunosuppressive therapy and tryptophan metabolism in COVID-19. In fact, tocilizumab has been proposed as a drug to counteract hyperinflammatory responses in ICU patients with COVID-19^35^. This possibility indicates that tryptophan-rich sources may be beneficial for COVID-19 patients^36^. The potential relevance of these observations confirms the impact of Trp and its metabolites against the severity of COVID-19, and our preliminary results suggest that unstimulated PBMCs analysed up to 90 days after SARS-CoV-2 infection show metabolism modulation through reactivation of the tryptophan pathway.

Another metabolism found to be significantly influenced by SARS-CoV-2 virus was arginine biosynthesis. Arginine is a semi-essential amino acid that can be obtained from the diet or produced in certain cells via the complete or partial urea cycle. In the urea cycle, arginase (Arg1) catabolizes arginine to produce urea, ornithine, polyamines and proline; when degraded by nitric oxide synthase (eNOS), the products are a large amount of NO and citrulline^37^. The ornithine used for citrulline production can originate from alanine and glutamate/glutamine reactions. In our results, arginine was not converted to ornithine. In fact, the latter was not fuelled by glutamine, which is a major substrate for citrulline production^38^. At the same time, the increased level of glutamine is in agreement with its role in boosting the immune system, especially by inhibiting inflammatory responses^39^. Indeed, adding enteral glutamine to normal nutrition in the early period of COVID-19 infection may lead to a shortened hospital stay and less need for ICU care^40^.

Conversion of arginine to citrulline rather than ornithine leads to release of NO. In the immune system, the NO produced in macrophages and neutrophils is necessary to kill invasive microorganisms (such as viruses, bacteria, and fungi) and activate immune cells in defence mechanisms^41,42^. Moreover, low plasma citrulline levels have previously been associated with acute respiratory distress syndrome in patients with severe sepsis^43^. Arginine is a substrate for nitric oxide (NO) production, inducing antiviral activity against RNA viruses, such as SARS-CoV-2^44^. In fact, decreased bioavailability of arginine and citrulline during inflammation can impede activation of T-lymphocytes and macrophage anti-inflammatory responses through a reduced capacity to support iNOS activity in SARS-CoV-2 (+) patients. This may have implications for vascular function and endothelial cell function, with reduced NO from immune cells attributed to immune suppression^45^. According to our results, accumulation of this amino acid in IgGm^+^ and consequent alteration of the urea cycle may enhance production of nitric oxide (NO)^44^. This latter mechanism is probably due to restoration of arginase (Arg1). It has been reported that Arg1 is located in the cytoplasm and is strongly expressed in the liver; it can regulate immune responses in addition to its metabolic role in the hepatic urea cycle. In fact, in humans, arginase is detected in peripheral blood mononuclear cells (PBMCs)^46^, and several studies highlight that Arg1 inhibits immunity against intracellular pathogens and represses T-cell-mediated inflammatory damage^47,48^. Arg1 up-regulation might be associated with a higher virus load in COVID-19 patients^49^. Since Arg1 can limit the bioavailability of L-arginine, inhibition of Arg1 can drive recycling of L-citrulline to generate L-arginine for NO production, contributing to development of antiviral immunity in IgGm^+^ . Interestingly, these results are in agreement with Xiao et al. who observed that supplementation with arginine markedly inhibits SARS-CoV-2-induced proinflammatory cytokine release by PBMCs, suggesting that serum arginine metabolism plays an ameliorative role in SARS-CoV-2-induced hyperinflammation^30^.

A significant proportion of L-arginine flux is attributable to the synthesis of creatine by the enzyme L-arginine:glycine amidinotransferase (AGAT). Generation of creatine is estimated to consume ~ 70% of labile methyl groups, with S-adenosyl-methionine (SAM) serving as the methyl donor^50^. This methylation demand may reduce methyl availability, causing SAM to be metabolized to S-adenosyl homocysteine (SAH) and finally to homocysteine, indirectly limiting methylation of the 5’ cap of the viral messenger RNA of coronavirus^22^. Therefore, it is not surprising that creatine kinase (an enzyme that catalyses conversion of creatine to create phosphocreatine) levels are higher in COVID-19 patients and are associated with more severe disease^51^.

In this study, for the first time, we provide new information to investigate antiviral defence mechanisms in memory leukocytes secreting in vitro IgG and SARS-CoV-2; however, as our research is very preliminary, there are several limitations. One limitation was the number of samples, and more samples are needed to follow up on the measured metabolites. Moreover, although our cohort included IgGm^+^ and IgGm^−^, there were no other biological samples from the same subjects at the time of acute infection, and their inclusion in future investigations will be informative and will improve our understanding of COVID-19. Finally, we used metabolomics analysis, which reflects the overall metabolic alterations of patients but cannot specifically reflect transcriptomic and proteomic changes; thus, future research is necessary to confirm these important results.

In conclusion, using the In-Cell ELISA Ig test, we detected more positive subjects who have encountered the virus compared to tests using conventional plasma or serum-based ELISA (Fig. 1). Moreover, mass spectrometry measurements revealed modulation of specific amino acid pathways associated with changes in leukocytes in response to COVID-19. This study demonstrates that the metabolism of patients at 60–90 days after infection determined by In-Cell ELISA is changed, providing novel methods for monitoring protection against the risk of SARS-CoV-2 reinfection and for examining whether the risk of reinfection changes over time.

## Supplementary Information

Supplementary Information 1.

## Data Availability

The datasets used and/or analysed during the current study are available from the corresponding author on reasonable request.
