# Supplementary figures and images for: An untargeted metabolomic approach to investigate antiviral defence mechanisms in memory leukocytes secreting anti-SARS-CoV-2 IgG in vitro
Source: Sci Rep. 2023 Jan 12;13:629. doi: 10.1038/s41598-022-26156-4 (PMC9835734; doi:10.1038/s41598-022-26156-4)

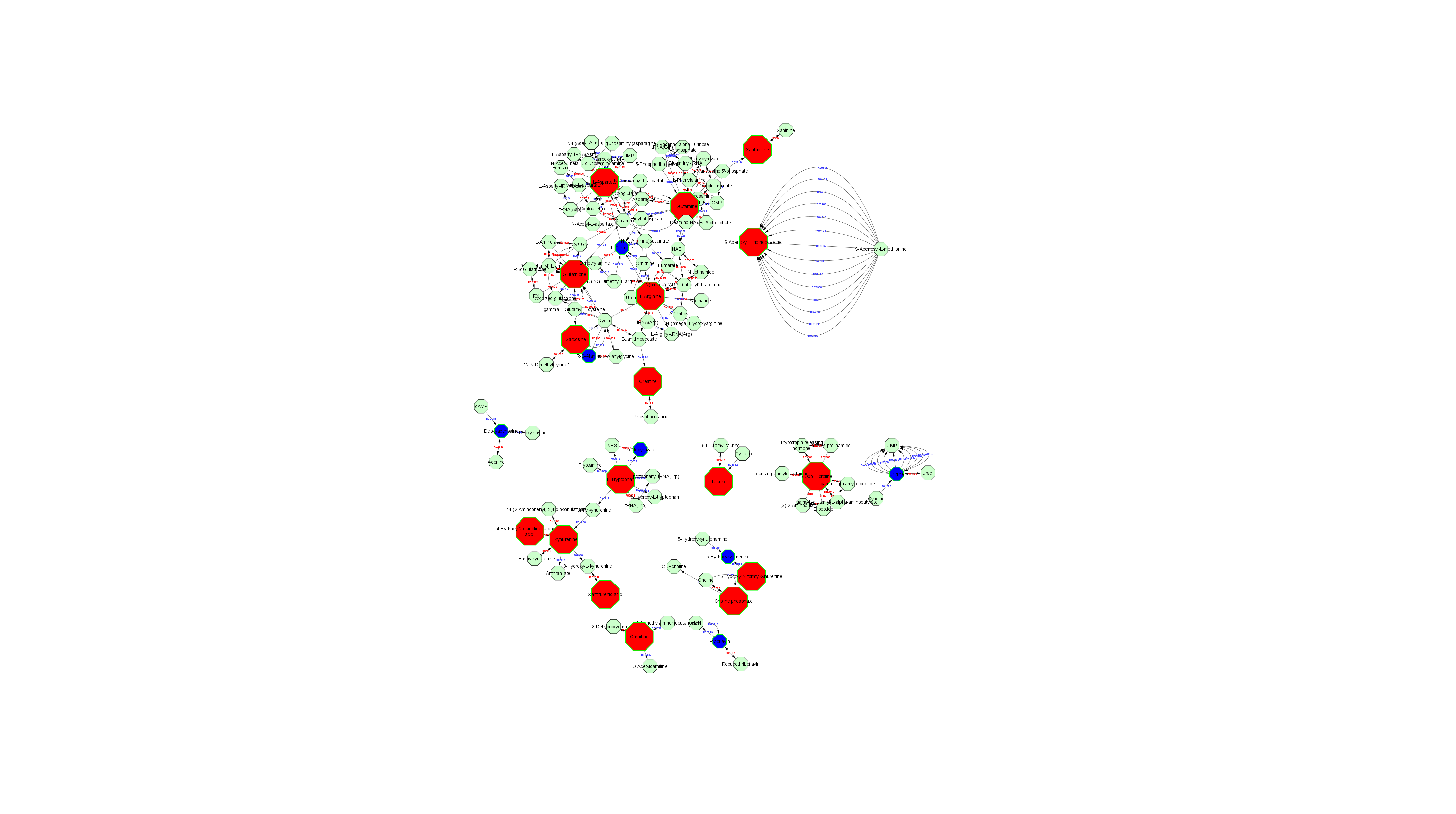

Supplement: Supplementary file 1 — Supplementary Information 1. [file 41598_2022_26156_MOESM1_ESM.png]
